# The protective effect of *Moringa oleifera* plant extract against glutamate-induced DNA damage and reduced cell viability in a primary retinal ganglion cell line

**DOI:** 10.7717/peerj.11569

**Published:** 2021-06-22

**Authors:** Musarat Amina, Ramesa Shafi Bhat, Abeer M. Al-Dbass, Nawal M. Musayeib, Rania Fahmy, Leen Alhadlaq, Afaf El-Ansary

**Affiliations:** 1 Department of Pharmacognosy, College of Pharmacy, King Saud University, Riyadh, Saudi Arabia; 2 Department of Biochemistry, College of Science, King Saud University, Riyadh, Saudi Arabia; 3 Department of Optometry, College of Applied Medical Sciences, King Saud University, Riyadh, r, Saudi Arabia; 4 Department of Ophthalmology, Faculty of Medicine, Cairo University, Cairo, Egypt; 5 College of Medicine, King Saud University, Riyadh, Saudi Arabia; 6 Central Laboratory, King Saud University, Riyadh, Saudi Arabia

**Keywords:** *Moringa oleifera*, Glutamate, Retinal Ganglion Cel, Comet assay, DNA damage

## Abstract

**Background:**

Glutamate excitotoxicity can cause DNA damage and is linked to many retinal and neurological disorders. In mammals, the visual signal from the eyes to the brain is conducted only by retinal ganglion cells (RGCs), which can be damaged by overstimulation of glutamate receptors.

**Methodology:**

We examined the protective effects of *Moringa oleifera* seed extract against glutamate-induced DNA damage in RGCs. RGCs cells were treated with 5, 10, 50, or 100 µg/ml of *M. oleifera* seed extract and glutamate separately and then assessed for DNA damage using the comet assay. We also evaluated the viability of the RGCs after both treatments using the MTT test. Additionally, RGCs were pretreated with *M*.* oleifera* seed extract (50 or 100 µg/ml) for 2 h before glutamate treatment (100 µg/ml) to determine the potential protective effects of *M. oleifera*. We performed a phytochemical analysis of the *M. oleifera* seed extract using standard reactions.

**Results:**

The *M. oleifera* seed extract was found to be rich in many phytochemicals. We observed a significant dose-dependent elevation in all comet assay variables in glutamate-treated RGCs, whereas *M. oleifera* seed extract treatments did not show any significant change in DNA integrity.

**Conclusion:**

*M. oleifera* seed extract demonstrates neuroprotective effects, which suggests it may help to prevent the development of many neurodegenerative disorders.

## Introduction

Glutamate is the major excitatory neurotransmitter in the mammalian brain (Zhou & Danbolt, 2014). More than 50% of neurons in the central nervous system use glutamate as their primary neurotransmitter and almost all thalamocortical, corticocortical, and corticofugal neurotransmission are mediated by glutamate (Nieuwenhuys, 1994; Fendt & Verstreken, 2017). Neuronal depolarization at presynaptic terminals releases glutamate, which is then recycled by excitatory amino acid transporters present in neurons and glia (Amara & Fontana, 2002; Javitt, 2004). Nearly 60% of brain energy metabolism is consumed in the reuptake and recycling of glutamate. This occurs through the conversion of glutamate to glutamine in glia, and the conversion back to glutamate via the action of neuronal glutaminase (Shulman, Hyder & Rothman, 2002; Massucci et al., 2013; Aldana et al., 2020). Although, glutamate plays a major role in neural development, differentiation, and plasticity, excess glutamate concentration can lead to excitotoxicity resulting in uncontrolled depolarization of neurons, finally leading to neuronal death (Mattson, 2003; Mattson, 2008; Hazell, 2007).

Glutamate plays a significant role in the visual system of retinal ganglion cells (RGCs) as it is the main excitatory neurotransmitter in these neurons (Massey & Miller, 1987; Massey & Miller, 1990; Thoreson & Witkovsky, 1999; Finlayson & Iezzi, 2010). Visual information captured by the eye is sent to the brain only through the optic nerve, which contains approximately 1.2 million RGC axons (Vrabec & Levin, 2007). RGCs are the meeting point for retinal information processing (D’Souza & Lang, 2020). Glutamate receptors are expressed by all types of retinal RGCs and N-Methyl-D-aspartate (NMDA) excitotoxicity is believed to result in RGC death in many retinal pathologies (Zhang & Diamond, 2009; Evangelho et al., 2017).

Preprocessed visual information is transmitted in the brain through RGC axons in sequences of action potentials. Mammalian RGCs express many neurotransmitter receptors and are able to receive excitatory and inhibitory input through chemical synapses; however, glutamate can damage RGCs through the overstimulation of glutamate receptors, namely, NMDA receptors (Lucas & Newhouse, 1957; Olney, 1982; Sisk & Kuwabara, 1985; Vorwerk et al., 1996). In mammals, many retinal diseases are linked to glutamate excitotoxicity since the visual signal from the eyes to the brain is only conducted by RGCs (Bringmann et al., 2013). Additionally, RGCs are unable to revive their axon if the optic nerve is damaged, which can result in optic neuropathies with complete visual loss (Bessero & Clarke, 2010). Therapeutic strategies to protect RGCs could provide a favorable effect to maintain the functions of these cells.

The products derived from various herbs and plants, being a rich source of bioactive compounds and multifunctional curing agents, are considered as relatively safe for the consumption. According to the Food and Agriculture Organization (FAO), around 70–80% of world’s population, particularly in developing countries, counts on herbal medicines for the cure and prevention of many diseases (Ekor, 2014). *Moringa oleifera* Lam. (Family Moringaceae), also known as drumkstick or horseradish tree, is considered as one of the most valued plant in the folk traditional medicine. It is an economical and easily available source of major essential nutrients and nutraceuticals, and has a remarkable ability to eradicate the malnutrition. The Moringa is often regarded as famous famine food due to its extreme resistance to arid and drought conditions owing to their tuberous roots (Saini, Sivanesan & Keum, 2016). Almost each part of this plant has been used for the preparation of useful medicine, nuteraceuticals, functional food, water purification and production of biodiesel (Kou et al., 2018). Various biological studies conducted on different extracts of *M. oleifera* have claimed that this plant has enormous potential to exhibit antioxidant, antimicrobial, anti-inflammatory (Gaafar et al., 2016), anticancer (Khor et al., 2018), hepatoprotective, antidiabetic (Muzumbukilwa, Nlooto & Owira, 2019), analgesic, antipyretic, immunomodulatory (Anudeep et al., 2016), gastroprotective, anti-ulcer (Adugba et al., 2018) cardiovascular, anti-obesity (Mabrouki et al., 2020), nootropic, diuretic, antiepileptic, anthelmintic, antiasthmatic, anti-allergic, anti-urolithiatic, local anesthetic, antidiarrhealand wound healing properties due to the presence of high amount of micronutrients and health-promoting phytochemicals (Bhattacharya et al., 2018). However, little is known about its neuroprotective effects against glutamate excitotoxicity (Iqbal & Bhanger, 2006; Igado et al., 2018).

Keeping in consideration the extensive chemical and pharmacological profile, we designed the present study to investigate the neuro-protective effect of *M. oleifera* seed extract in ameliorating the genotoxic and cytotoxic effects of glutamate-induced excitotoxicity in a primary RGC line through the use of COMET assay and cell viability techniques respectively.

## Materials & Method

### Plant material

The seeds of the *Moringa oleifera* were purchased from the local market of the Riyadh, Saudi Arabia. Sample material was identified and authenticated by Prof. Dr. Mohamed Yousef of the Pharmacognosy Department, College of Pharmacy at King Saud University, Saudi Arabia. A voucher specimen (MO-5302) has been kept in the herbarium of the same department. Seeds were cleaned of dirt, washed and dried at room temperature. The dried seeds were finely powdered by using domestic blender, collected in plastic bags and placed in cold place at 4 °C, prior to use.

### Extraction of plant samples

The powdered seeds (1 kg) of *M. oleifera* were soaked in methanol (3 L) with continuous shaking at 2 h intervals for 78 h at room temperature to obtain maximum extraction of bioactive constituents (Do et al., 2014; Dhawan & Gupta, 2017). The obtained extract was filtered through Whatman No. 1 filter paper and centrifuged to remove the suspended material. The extraction process was repeated thrice under the similar conditions. All the organic extracts were pooled and freed from organic solvent on rotaevaporator at 40 °C under reduced pressure to yield a dark brown residue (69.8 g). The dried residues were transferred to glass tubes, tightly stoppered, and stored at 5 °C until further use.

### Phytochemical screening *M.olifera* seed extract

Chemical tests were performed for the phytochemical analysis of compounds present in the extract. These tests include Borntrager’s test for anthraquinones, Shinoda test for phenols, Ferric chloride test for flavonoids, Libermann–Burchard test for steroids–triterpenoids, lead acetate test for tannins, Dragendorff’s test for alkaloids, Rosenthaler test for saponins, and Keller–Killiani test for cardiac glycoside (Brain & Turner, 1975; Harborne, 1998; Samejo et al., 2013).

### Quantitative analysis of *M.olifera* seed extract

Spectrophotometric assay was used to perform the phytochemical analysis of methanolic extract of *M.olifera* seed by obeying Onwukeame et al. method with little modifications (Onwukeame, Ikuegbvweha & Asonye, 2007). The total phenolic composition in *M.olifera* extract was calculated by plotting a graph of gallic acid standard as previously mentioned (Luximon-Ramma et al., 2002). Briefly, 0.120 mL of seed extract was mixed with 0.5 mL of distilled water, followed by the slow addition of 0.12 mL of Folin–Ciocalteu reagent and incubated at ambient temperature for 5 min. Afterwards, 7% of sodium bicarbonate solution (1.20 mL) was added to the reaction mixture and volume made up two mL with distilled water and incubated for 1 h at ambient temperature. After 1 h incubation, the absorbance was recorded at 760 nm. The results were calculated as µg of gallic acid equivalents/mg of the seed extract (GAEs). However, the measurement of flavonoid content in the seed extract of *M.olifera* was performed by using a quercetin reference curve as previously described by Ghosh et al. (Ghosh et al., 2012). Briefly, one mL of seed extract of *M.olifera* was mixed with one mL of 2% aluminum chloride and incubated for 30 min at room temperature. After 30 min incubation, the absorbance was measured at 368 nm. The results were calculated as µg of quercetin equivalents (QEs)/mg of seed extract. Flavonols content in the seed extract was estimated by using rutin as a reference. The method is based on the complex formation displaying absorption at 440 nm (Grubesic et al., 2005). The results were determined as µg of rutin equivalents/mg of seed extract. The content of tannin in *M.olifera* seed extract was determined by applying Folin-Denis procedure (Oyaizu, 1986). The method is based on the formation of a precipitate upon treatment with casein showing absorption at 720 nm. The results were calculated as µg of tannic acid equivalents/mg of seed extract.

### Antioxidant activity of methanol extract of *M.oleifera*

Two diverse radical scavenging assays, DPPH (1, 1-diphenyl- 2-picrylhydrazyl) and ABTS (2, 20-azino-bis [3-ethylbenzothiazoline-6-sulphonic acid]) free radical scavenging were applied to examine the antioxidant potential of methanol seed extract of *M.oleifera*.

### DPPH free radical scavenging activity

The DPPH free radical scavenging activity of the seed extract of *M.oleifera* and butylated hydroxytoluene (BHT) (Sigma-Aldrich, St. Louis, Missouri, USA) as the reference standard, was determined by obeying standard procedure (Mensor et al., 2001). Briefly, 0.1 mM DPPH reagent (Sigma-Aldrich, St. Louis, Missouri, USA) in methanol and three different concentrations (25, 50, 100 µgmL^−1^) of *M.oleifera* extract or BHT in methanol were prepared prior to the experiment. The reaction mixture was initiated by adding 50 µL of DPPH solution and varied concentration of *M.oleifera* extract and BHT to each well of 96-well microplate. The whole reaction mixture was subjected to continuous shaking using orbital shaker for 30 min under dark conditions. After 1 h incubation, the absorbance of the reaction mixture was measured at 593 nm using a UV-vis spectrophotometer (Multiskan GO; Thermo Scientific, Waltham, MA, USA), with methanol as a blank. All the measurements were performed in triplicates. The scavenging abilities of *M.oleifera* seed extract and BHT on DPPH free radicals was calculated by applying the following equation:

(1) \begin{eqnarray*}\mathrm{DPPH~ free~ radical~ scavenging}(\text{%})= \frac{Cr-Tr}{Ct} \times 100\end{eqnarray*}

Where Cr and Tr is the absorbance of control and the test samples, respectively

### ABTS free radical scavenging activity

A standard method described by Re et al. (1999) was followed to evaluate the ABTS free radical scavenging potential of the seed extract of *M.oleifera* and BHT taken as a reference standard. Briefly, two stock solutions of 7.4 mM ABTS and 2.6 mM potassium persulfate (Sigma-Aldrich, St. Louis, Missouri, USA) were prepared in methanol individually. To prepare the working solution of ABTS, equivalent amounts of stock solutions were mixed and kept undisturbed for 16 h under dark conditions at room temperature. In a 96-well microplate, 135 µL of the ABTS working solution and different concentration (25, 50, 100 µgmL^−1^) of *M.oleifera* extract and BHT to each well. The reaction was mixed and allowed to stand in dark for 2 h at ambient temperature. The UV–Vis spectrophotometer was used to record the absorbance of the reaction mixture at 743 nm, with methanol as a control. All the measurements were carried out in triplicates. The scavenging abilities of *M.oleifera* seed extract and BHT on DPPH free radicals was calculated by applying the following Eq. (1).

### Isolation and purification of RGCs

The Retinal cells were obtained with the help of expert in Animal Reproduction Research Institute (ARRI)-Kafr Nassar, Al Haram, Giza Governorate, Egypt. Six four-day- old Sprague-Dawley rats were used to isolate RGCs. The rats were kept in standard housing conditions with 12 h light/dark cycle in a temperature-controlled room with free access to food and water. Rats were euthanized by using CO_2_ inhalation followed by cervical dislocation. Eyes were dissected out, and excess muscle and connective tissue were removed in ice cold phosphate-buffered saline (PBS). RGCs were purified and stored in isotonic buffer solution  containing inorganic salts, glucose, papain, and DNase I for half an hour. Papain activity was stopped using ovomucoid solution. A 40 µm mesh filter was used to obtain the single-cell suspensions using a filtering process. Negative and positive cell selection was performed using rabbit anti-rat macrophage/Thy-1 antibody-coated Petri dishes. Panning plates were incubated overnight at 4 °C and rinsed 3 times with balanced salt solution . Poly-D-Lysine solution was added and cells were incubated overnight at room temperature. Dried cells were incubated with Mouse laminin for 120 min at 37 °C. Finally RGCs were seeded on PDL- and laminin-coated coverslips in RGC growth medium at 37 °C in a 5% CO_2_ incubator. Half quantity of medium was replaced on the third day. The protocol of this work was approved by ethical committee, King Saud University with approval number EC Ref No.: 4/67/352670.

### Cell treatment

Pure RGCs were divided into three groups depending upon the treatment: control (without treatment); *M. oleifera* treatment (5, 10, 50, or 100 µg/ml of seed extract); glutamate treatment (5, 10, 50, or 100 µM glutamate for 48 h). Finally, cells individually pretreated with extracts (50, or 100 µg/ml) were exposed to 100 µM glutamate for 48 h.

### Comet assay

The method described by Singh et al. (1988) was uperformed for the comet assay. Cells were treated with test material for 24 h in Petri dishes. Cells were trypsinized (0.1% for 4 min), suspended, and centrifuged for 10 min at 800 rpm. Afterward, 600 µl of 0.8% low-melting agarose was added to the cell suspension and relocated to pre-coated agarose slides. The coated slides were dipped in lysis buffer (0.045 M TBE, pH 8.4, having 2.5% SDS) for 20 min. The slides were placed on a gel electrophoresis and covered with ice-cold alkaline solution (300 mM NaOH and 1mM Na2 EDTA, pH 13) in the dark at 0 °C for 20 min, before the electrophoretic run. The electrophoresis settings were 2 V/cm for 20 min and 100 milliampere (mA). Ethidium bromide (20  µg/ml at 4 °C) was used for staining. DNA fragment migration patterns of 100 cells for each dose level were evaluated with a fluorescence microscope. DNA damage was measured as tail length (TL = distance of DNA migration from the center of the body of the nuclear core) and tail intensity of DNA (TI = % of genomic DNA that migrated during the electrophoresis from the nuclear core to the tail).

### Determination of cell viability

The MTT test was used to measure cell viability. Absorbance was recorded at 490 nm using a microplate reader. The results are presented as a percentage of control (untreated cells) or as a percentage of glutamate excitotoxicity.

### Statistical analysis

Data were analyzed using the Statistical Package for the Social Sciences (SPSS, Chicago, IL, USA). Results are presented as mean ± standard error (SEM). All statistical comparisons among groups used one-way analysis of variance complemented with Dunnett’s test for multiple comparisons. Significance was considered as *p* < 0.05.

## Results

### Phytochemical description of the extracts

The phytochemicals present in the crude extract of *M. oleifera* seeds are listed in Table 1. The methanol seed extract of M.olifera showed the presence of a variety of bioactive constituents, including phenolics, flavonoids, flavonols, tannins, and steroids. These bioactive components were qualitatively screened by phytochemical analysis and might be responsible for the neuroprotective effects. The content of secondary metabolites such as phenols, flavonoids, flavonols and tannins in the methanol seed extract of *M.olifera* was summarized in Table 2. The estimation of phenolics, flavonoid, flavonol and tannin content in the *M.olifera* seed extract was determined by using gallic acid, quercetin, rutin and tannic acid respectively, as standards. The obtained results revealed that the seed extract of *M.olifera* contains total phenolic, flavonoid, flavonol and tannin content in the range of 78.59 ± 0.98 µg mL^−1^, 112.23 ± 1.1 µg mL^−1^, 4.98 ± 0.56 µg mL^−1^ and 23.73 ± 0.65 µg mL^−1^, respectively.

### Antioxidant activity of methanol extract of *M.oleifera*

The antioxidant potential *M.oleifera* seed extract was investigated according to assays performed in various radical scavenging studies, i.e., the DPPH free radical scavenging and ABTS free radical scavenging assays. DPPH and ABTS^•+^ cation radicals are reactive against most of the antioxidants and their decolorization indicates the abilities of antioxidants to donate hydrogen atoms or electrons to inactivate the radical species. The IC_50_ values of the extract for both DPPH radical scavenging and ABTS^•+^ cation radical scavenging are presented in Table 3. The obtained results revealed that the *M.oleifera* seed extract showed similar activity trend towards both the assays. *M.oleifera* seed extract displayed the lowest IC_50_ values for both DPPH (18.68 ± 5.54) and ABTS^•+^ (21.25 ± 2.22) radical scavenging assays and hence exhibited the highest radical scavenging potential. There was no significant difference (*p* ≥ *0.05*) in the scavenging effect of DPPH and ABTS. The strong DPPH and ABTS^•+^ radical scavenging effects of seed extract of *M.oleifera* may be credited to the presence high phenolic and flavonoid contents in extract.

**Table 1:** Phytochemical screening of crude methanol extracts of *M. oleifera* seeds.

Identified phytoconstituents	*M. oleifera*
Phenolics	+++
Flavonoids	+++
Tannins	++
Triterpenoids	+++
Coumarins	++
Cardiac glycosides	++
Anthraquinones	+
Alkaloids	+

**Notes.**

- absent + low intensity ++ medium intensity +++ strong intensity

**Table 2:** Phytochemical estimation of phenolic, flavonoids, flavonol, tannins content in methanol extract of *M.olifera* seed extract.

S.No	Phytochemical components	*M.olifera* seed extract
		Absorbance (nm)	Content (µg mL^−1^)
1	Total phenolic	0.224 ± 0.65	78.59 ± 0.98
2	Total flavonoids	0.446 ± 0.02	112.23 ± 1.1
3	Total flavonols	0.124 ± 0.14	4.98 ± 0.56
4	Total tannins	0.014 ± 0.002	23.73 ± 0.65

### Comet assay

**Figure 1:**
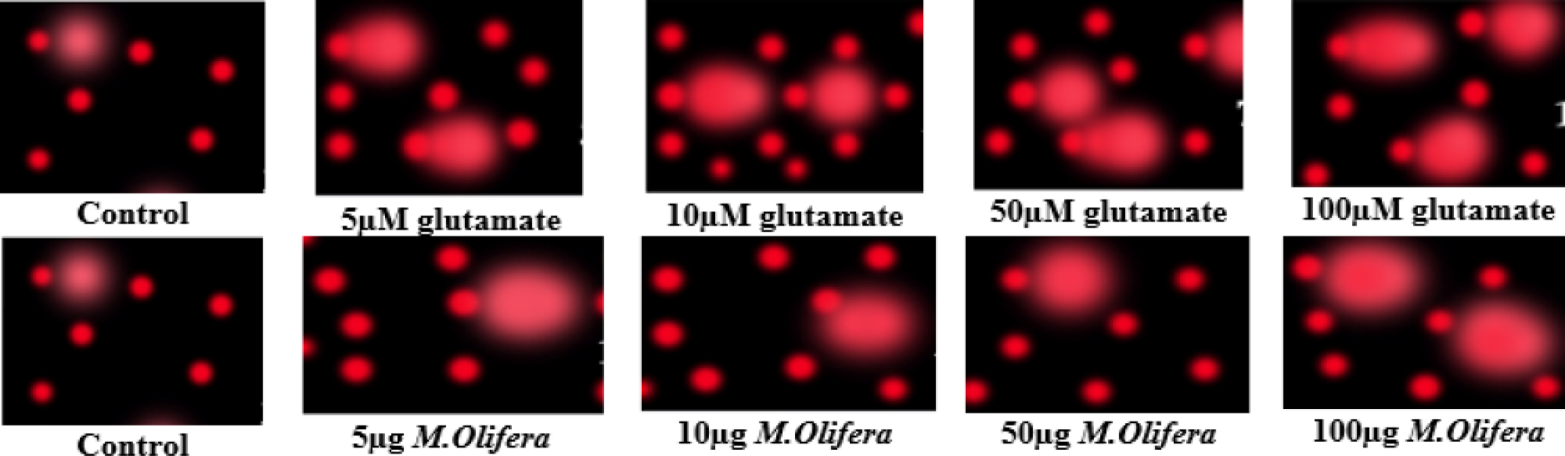
Measurements of glutamate-induced DNA damage by comet assay in control, glutamate-treated, and *M. oleifera*-treated RGCs.

**Figure 2:**
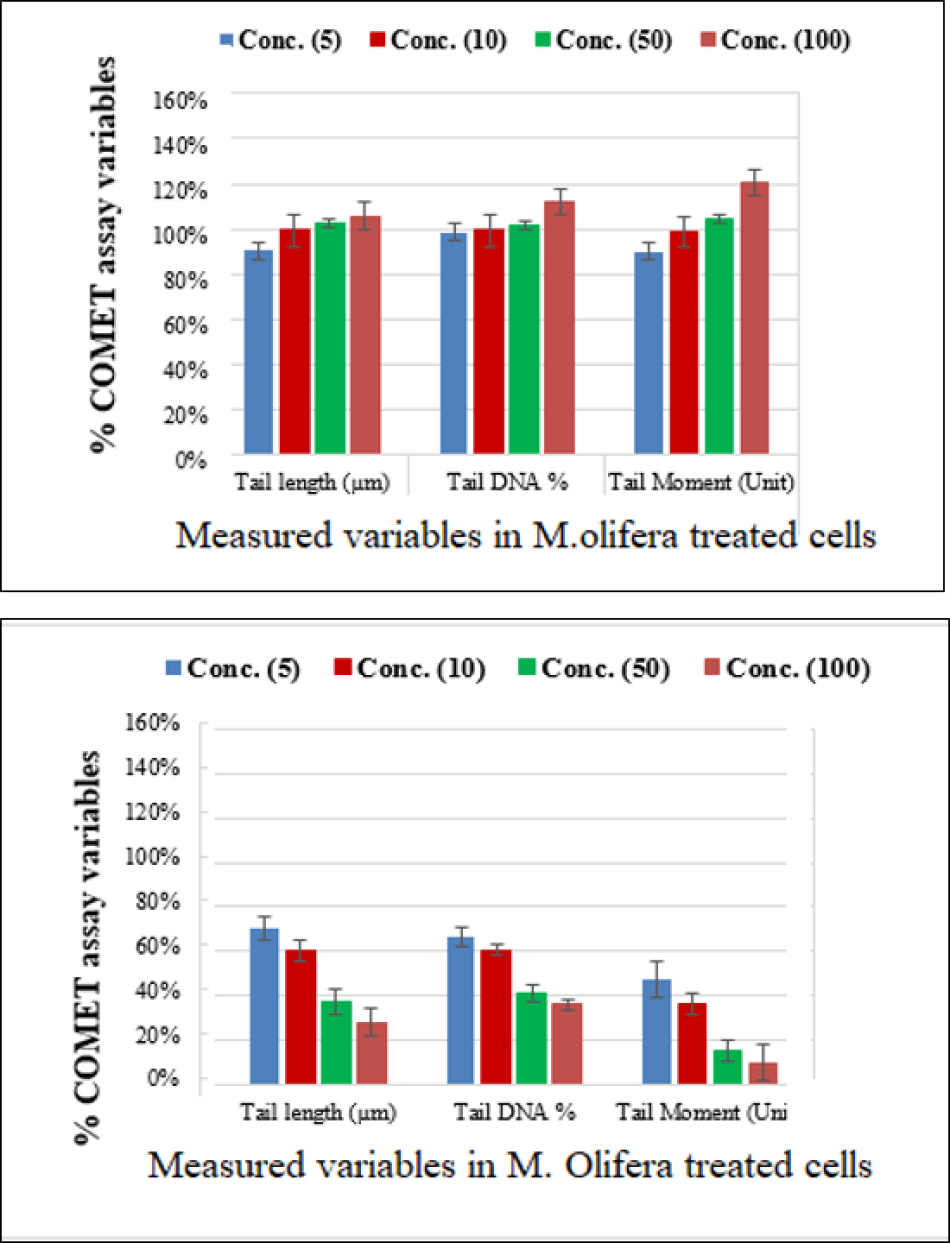
Percentage change in comet assay variables of *M. oleifera*-treated RGCs with control untreated cells and glutamate excitotoxic cells.

Protective effect of *M. oleifera* seed extract against glutamate-induced DNA damage in a primary RGC was evalvated by results of Comet assay. Assessment of the genotoxic activity of glutamate and antigenotoxic activities *M. oleifera* seed extract were estimated by the parameter related to percentage of tailed DNA in treated cells as compared to control. Table 4 presents the comet assay measured variables of cells in all groups. Glutamate was found to be significantly toxic in a dose-dependent manner for RGCs as compared to control untreated RGCs*.* Glutamate-induced DNA damage in RGCs is shown in Figs. 1 and 2. We noticed negligible damage with *M. oleifera* seed extract as compared with glutamate (Figs. 1–2).

**Table 3:** The IC_50_ values for the DPPH and ABTS^⋅+^ radical scavenging activities of the seed extract of *M. olifera*.

**Sample**	**Concentration** (µgmL^−1^)	**DPPH radical scavengingIC**_50_ (µg/ml)	**ABTS radical scavengingIC**_50_ (µg/ml)
*M.oleifera* seed extract	25	42.03 ± 0.65	45.42 ± 2.2
	50	34.13 ± 1.02	38.12 ± 0.98
	100	18.68 ± 5.54	21.25 ± 2.22
BHT	100	10.65 ± 0.65	13.98 ± 1.01

**Table 4:** Comet assay-measured variables in *M. oleifera* seed extract-treated and glutamate-treated retinal cells.

COMET assay variables	Treatment	Concentration			
		5 ug/ml	10 ug/ml	50 ug/ml	100 ug/ml
Tail length (µm)	Control	1.23 ± 0.09	1.23 ± 0.09	1.23 ± 0.09^a^	1.23 ± 0.09
	Glutamate	1.57 ± 0.13^a^	2.03 ± 0.22^a^^#^	3.36 ± 0.32^a^^#^	4.62 ± 0.41^a^^#^
	M. olifera	1.11 ± 0.06	1.22 ± 0.06	1.26 ± 0.07^#^	1.30 ± 0.08^a^
Tail DNA (%)	Control	1.24 ± 0.16	1.24 ± 0.16	1.24 ± 0.16	1.24 ± 0.16
	Glutamate	1.83 ± 0.12^a^	2.03 ± 0.11^a^^#^	3.06 ± 0.15^a^#	3.85 ± 0.58^a^^#^
	M. olifera	1.22 ± 0.05	1.23 ± 0.03	1.26 ± 0.05	1.39 ± 0.03^#^
Tail Moment (Unit)	Control	1.51 ± 0.10	1.51 ± 0.10#	1.51 ± 0.10#	1.51 ± 0.10
	Glutamate	2.88 ± 0.41^a^^#^	4.12 ± 0.44^a^^#^	10.32 ± 1.49^a^^#^	17.96 ± 4.09^a^^#^
	M. olifera	1.36 ± 0.11	1.49 ± 0.07#	1.58 ± 0.08#	1.82 ± 0.14^#^

**Notes.**

a *p* < 0.001, value between each group and the control group.

# *p* < 0.001 value between all groups.

All groups were compared using one-way ANOVA with Dunnett test (Multiple Comparisons) to compare each group with the control group Kruskal-Wallis test was used to compare all groups using parametric data. Mann–Whitney test was used to compare each group with the control group for non-parametric data.

### Cell viability

**Figure 3:**
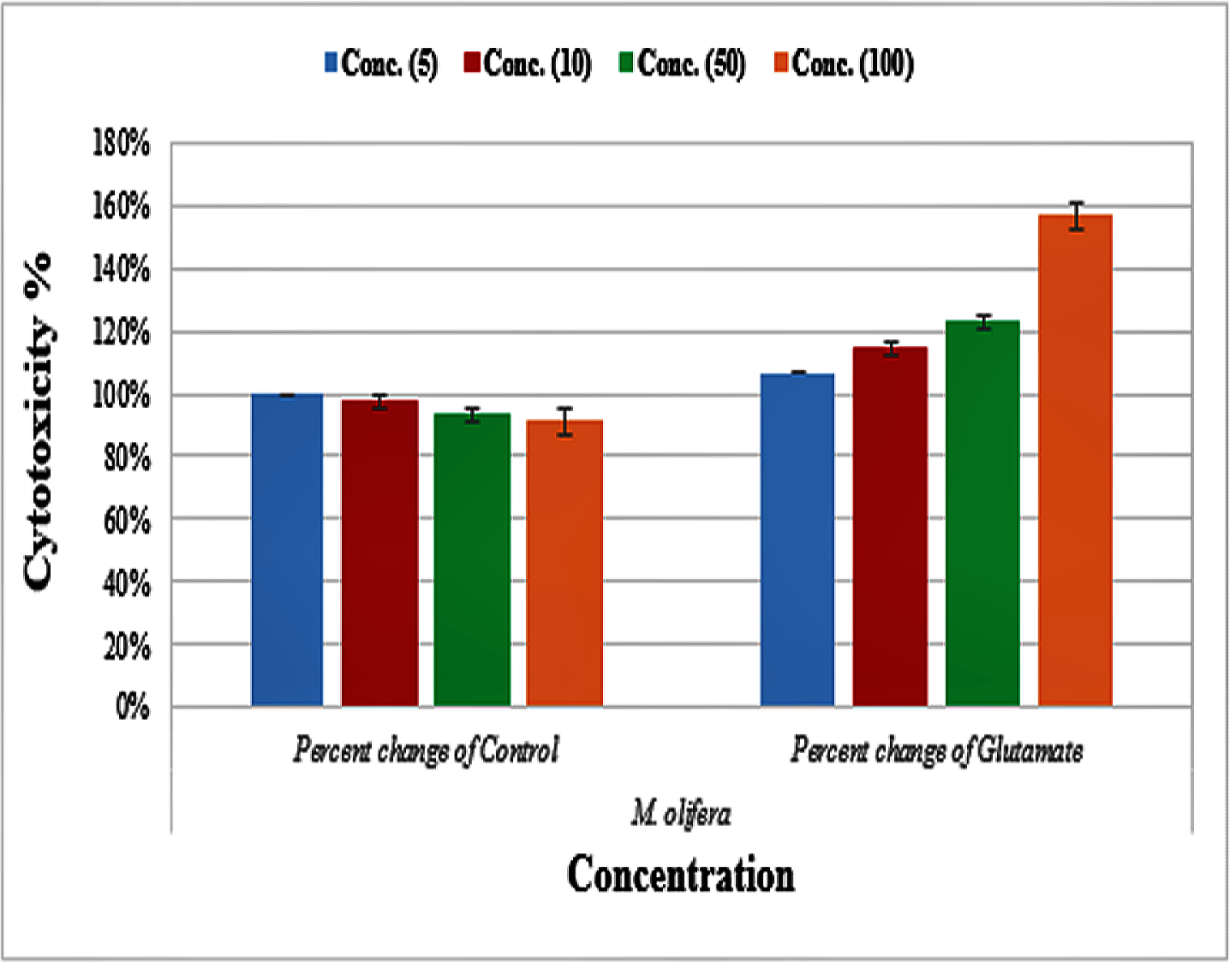
Percentage change of cell viability with various *M. olifera* concentrations compared to the control and glutamate groups.

**Figure 4:**
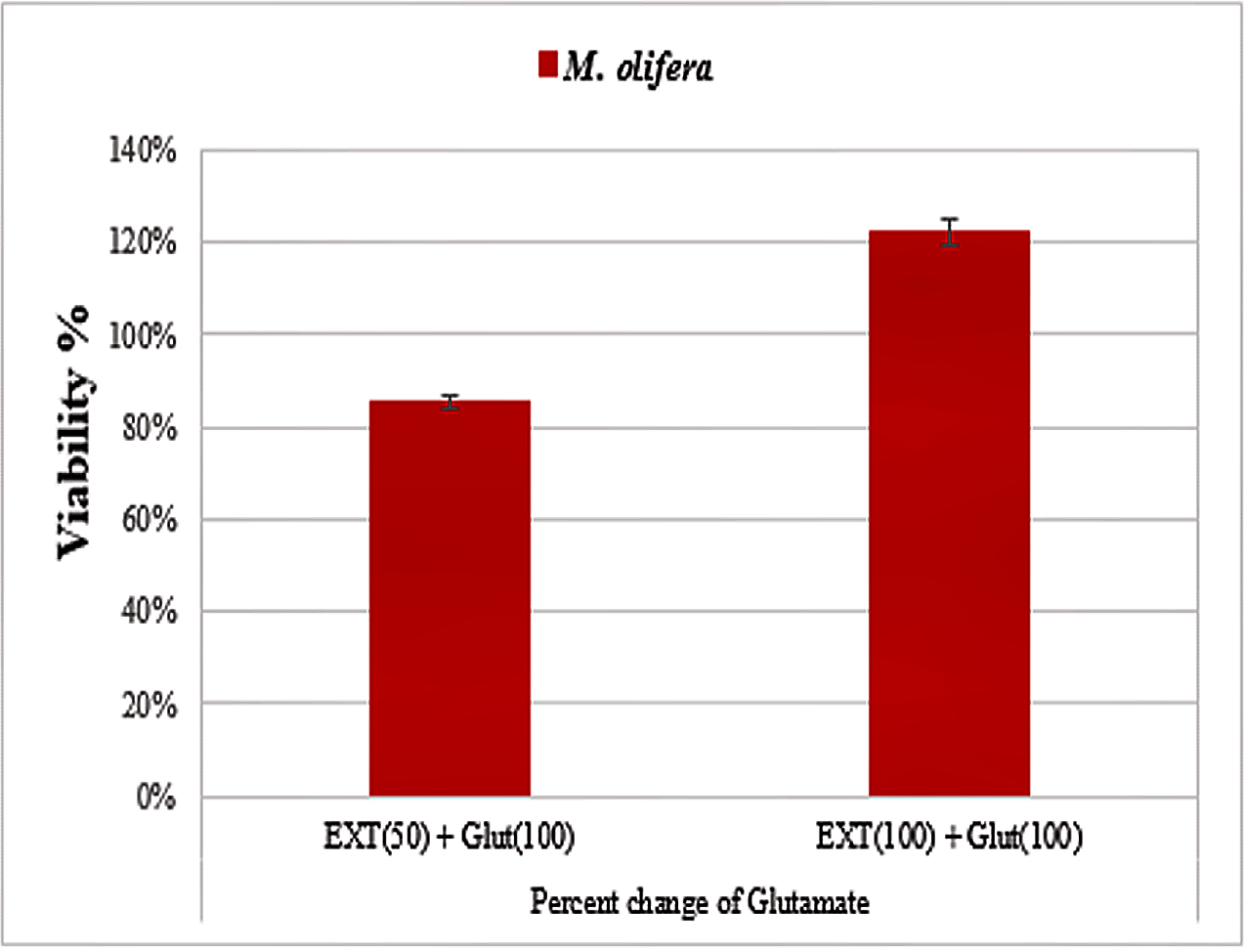
Percentage change in *M. oleifera-*treated cells (EXT)** compared to glutamate on cell viability.

Protective effect of *M. oleifera* seed extract against glutamate-induced neurotoxicity in a primary RGC was done by calculate cell viability and proliferation through MTT assay. In this assay metabolic reduction of the soluble 3-(4,5-dimethylthiazol-2-yl)-2,5-diphenyltetrazolium bromide (MTT) salt to insoluble colored formazan product by mitochondria dehydrogenase of live RGC cells was measured spectrophotometrically at 480 nm. The amount of formazan dye formed in cells treated with glutamate and *M. oleifera* seed extract was directly correlates to the number of viable cells. Results of cytotoxic effects on RGC proliferation after treatment are presented in Table 5 and Fig. 3. *M. oleifera* seed extract showed negligible toxicity compared to glutamate and showed a dose-dependent reduction in cell viability. The inhibition of cell proliferation was most obvious at the 100 µM concentration, which suggests dose-dependency. Cell viability of the seed extract-treated RGCs was significantly different compared to glutamate intoxicated cells. Cyto-protective effects of the seed extract were evaluated by pretreating cells with the extract before exposing the RGCs to glutamate. These results are shown in Table 6. RGCs were pretreated with 50 and 100 µg/ml of the seed extract for 2 h before exposing the cells to 50 µM and 100 µM of glutamate. We observed a remarkable increase in cell viability (Table 6 and Fig. 4).

## Discussion

The seed extract was found rich in flavonoid and phenolic components due to the presence of free hydroxyl (OH) and carboxyl (CO) groups in their structure. Recently, the implications of excitotoxicity as an agent of human diseases have been highlighted and given special attention as etiological mechanisms of many diseases reveals a critical need for developing anti-excitotoxic neuroprotective strategies (Wu & Tymianski, 2018; Fern & Matute, 2019; Hardingham, 2019; Ge et al., 2020). The present study demonstrates the protective potential of *M. oleifera* seed extract against glutamate-induced DNA damage in RGCs. To evaluate possible DNA damage induced by glutamate in RGCs, we used the comet assay as this technique has been successfully applied in cell cultures and neurons to explore toxicity (Chao et al., 1999; Lamarche et al., 2003; Signorini-Allibe et al., 2005). We observed a significant increase in DNA damage in cells treated with glutamate. This result was expected since DNA damage in neurons is an early sign of excitotoxicity (Didier et al., 1996; Gwag et al., 1997). Many severe ocular diseases occur due to increased glutamate levels causing retinal neurodegeneration and RGC death (Bringmann et al., 2013; Fernandez-Sanchez et al., 2017; Xia & Rizzolo, 2017; Blanco et al., 2017). Neurons tend to show reversible nuclear oxidative DNA damage in response to glutamate receptor activation (Yang et al., 2010).

**Table 5:** Comparison with control group (Viability); glutamate group (Viability).

Treatment	Concentration			
	5 ug/ml	10 ug/ml	50 ug/ml	100 ug/ml
Control	1.00 ± 0.00	1.00 ± 0.00	1.00 ± 0.00	1.00 ± 0.00
Glutamate	0.94 ± 0.02	0.85 ± 0.02	0.76 ± 0.01	0.58 ± 0.03
M. olifera	1.00 ± 0.00^#^	0.97 ± 0.02a^#^	0.93 ± 0.02a^#^	0.91 ± 0.04a^#^

**Notes.**

a Significant difference compared with the control group at 0.05 level.

# Significant difference compared with the glutamate group at 0.05 level and 0.01 level.

All groups were compared using one-way ANOVA test with Dunnett test (Multiple Comparisons) to compare each group with the control group. For parametric data, Kruskal-Wallis test was used to compare all groups. For non-parametric data, Mann–Whitney test was used to compare each group with the control group.

In the present study, the genotoxic potential of glutamate was clearly demonstrated by the dose-dependent elevation in the comet variables, indicating the extent of DNA damage in the neuronal cell culture (Table 4). The RGCs were exposed to 5, 10, 50, or 100 µg/ml glutamate and all doses caused significant DNA damage with higher concentrations showing greater damage. Many previous studies have described toxic concentrations of glutamate with a similar trend (Mattson, Dou & Kater, 1988; Choi, 1992; Mattson, 2003; Balazs, 2006). A high level of glutamate in cells can prompt DNA damage and cell death in rat primary cerebral cortical cultures (Yang et al., 2011). High glutamate levels can induce mitochondrial Ca2+ uptake with raised mitochondrial respiration, which can increase superoxide and other genotoxic free radicals and trigger DNA damage and neuronal death (Sengpiel et al., 1998; Chinopoulos et al., 2000; Kruman et al., 2000; Culmsee et al., 2001). More recent studies have indicated that in response to glutamate excitotoxic level, Ca^2+^ and oxidative flows can interact in endoplasmic reticulum-mitochondrial signaling and trigger mitochondrial membrane permeabilization followed by the activation of caspase-dependent or caspase-independent apoptosis signaling (Tajeddine, 2016; Hempel & Trebak, 2017; Humeau et al., 2018).

**Table 6:** Comparison with glutamate group (Viability).

Parameters	Extracts	Mean ± S.D
Extract (50) + Glut (100)	Glutamate	0.76 ± 0.01
	M. olifera	0.65 ± 0.01^#^
Extract (100) + Glut (100)	Glutamate	0.58 ± 0.03
	M. olifera	0.71 ± 0.02^#^

**Notes.**

# Significant difference compared with the glutamate group at 0.01 level.

One-way ANOVA test with multiple comparisons (Dunnett test) was used.

After exposing neurons with the same concentration (5, 10, 50, or 100 µg/ml) of *M. oleifera* seed extract, DNA damage values were slightly different from controls even at the higher concentration, which suggests low toxicity of the extract. Triterpenoids, cardiac glycosides, anthraquinones, and flavonoids that are present in the seed extract are known for antioxidant and free radical scavenging potential (Table 1). The presence of such phytochemicals suggests that the extract should be evaluated for its health-protective potential. Studies by Jahan et al. (2018) have also shown strong antioxidant and free radical scavenging activities of *M. oleifera* seed extract. Glutamate caused a marked decrease in the viability of RGCs, especially at higher concentrations, whereas *M. oleifera* seed extract showed almost negligible effects even at higher concentrations (Table 5). Many studies show healing effects of *M. oleifera* seed extracts through their antioxidant and detoxification mechanisms (Pakade, Cukrowska & Chimuka, 2013; Jahan et al., 2018). *M. oleifera* has a long history in Ayurveda medicine of treating many central nervous system diseases (Hannan et al., 2014). *M. oleifera* extract has been found to increase neuronal cell viability with minimal cellular injury. It can also extend branches in neurons, can modulate axonal development, and promote synaptogenesis (Hannan et al., 2014). We observed neuroprotective effects of *M. oleifera* when cells were pretreated with 50 and 100 µg/ml of *M. oleifera* seed extract before glutamate treatment (Table 6, Fig. 3). These results suggest that *M. oleifera* seeds could be further explored for the treatment of neurodegenerative disorders. *M. oleifera* seed, being rich in phytochemicals (Table 1) can be linked to its ameliorating effect against glutamate excitotoxicity in RGCs as oxidative stress and detoxification mechanisms are considered among the main etiological factors in neurological disorders.

## Conclusions

Under the conditions employed in the current study, *M. oleifera* extract demonstrated chemoprotection against the genotoxic and cytotoxic effects of glutamate on RGC.

##  Supplemental Information

10.7717/peerj.11569/supp-1 Supplemental Information 1 Raw data

10.7717/peerj.11569/supp-2 Supplemental Information 2 Antioxidant raw data

## Additional Information and Declarations

### Data Availability

The following information was supplied regarding data availability: The raw data are available in the Supplemental Files.
